# Seeing the Unseen: Artificial Intelligence-Assisted Detection of Subtle Colorectal Adenomas During Colonoscopy

**DOI:** 10.7759/cureus.104150

**Published:** 2026-02-23

**Authors:** Kyungchul Kim, Gene C Lim, Alfredo Noches-Garcia

**Affiliations:** 1 General Surgery, Rockingham General Hospital, Perth, AUS

**Keywords:** adenoma detection, artificial intelligence (ai), colonoscopy, computer-aided detection, high-grade dysplasia

## Abstract

Colonoscopy is central to colorectal cancer (CRC) prevention, with adenoma detection rate (ADR) serving as a key quality indicator. Artificial intelligence (AI)-based computer-aided detection (CADe) systems have been developed to assist endoscopists by highlighting subtle mucosal abnormalities during withdrawal. We report the case of a 67-year-old male who underwent AI-assisted colonoscopy for a positive faecal occult blood test, during which CADe prompted the detection and resection of 30 additional polyps not prospectively recognised during conventional white-light inspection. Histopathological analysis demonstrated predominantly tubular adenomas with low-grade dysplasia, as well as a tubulovillous adenoma with focal high-grade dysplasia. This case highlights the potential of CADe to lower the visual threshold for adenoma detection while also illustrating the associated procedural burden and clinical uncertainties surrounding extensive AI-guided lesion detection in routine practice.

## Introduction

Colorectal cancer (CRC) remains a major global health burden and is a leading cause of cancer-related morbidity and mortality. Worldwide, CRC is the third most commonly diagnosed malignancy and the second leading cause of cancer-related death [1]. CRC develops through a multistep process driven by the accumulation of genetic and epigenetic alterations within the colonic epithelium, progressing from adenoma formation to invasive carcinoma [1].

Colonoscopy plays a central role in CRC screening and prevention by enabling the detection and removal of premalignant lesions. Its effectiveness is closely linked to adenoma detection rate (ADR), a validated quality indicator inversely associated with post-colonoscopy CRC risk [2]. Even modest improvements in ADR translate into meaningful reductions in subsequent CRC incidence and mortality [2].

Adenomas with advanced histological features, including villous architecture and high-grade dysplasia, are recognised as premalignant lesions associated with increased malignant potential. Resection of such lesions has been shown to reduce CRC incidence and mortality, and they are therefore considered high-risk findings in post-polypectomy surveillance guidelines [3-5].

Recent advances in artificial intelligence (AI), particularly computer-aided detection (CADe) systems, have introduced a novel adjunct to colonoscopy. CADe systems analyse endoscopic video streams in real time, acting as an additional observer that highlights suspected mucosal abnormalities during withdrawal [6]. Unlike human operators, CADe is not subject to fatigue or perceptual bias and may reduce dependence on endoscopist experience, particularly for subtle or diminutive lesions [7,8]. Meta-analyses report a relative increase in ADR of approximately 20% with CADe, alongside reduced adenoma miss rates and minimal prolongation of withdrawal time in controlled trials [9].

However, the clinical implications of increased lesion detection remain incompletely understood. While CADe consistently improves detection of diminutive adenomas, its impact on advanced neoplasia and sessile serrated lesions appears more limited [7,9]. Enhanced detection may also increase identification and resection of non-neoplastic or clinically indolent lesions, raising concerns regarding overdiagnosis, procedural risk, pathology burden, and healthcare costs [9]. Additional concerns include over-reliance on AI and unintended effects on endoscopist behaviour.

This report describes a patient who underwent an AI-assisted colonoscopy using the Olympus ENDO-AID CADe system (Olympus Corporation, Tokyo, Japan), highlighting both the potential benefits and unintended consequences of extensive AI-guided lesion detection in routine clinical practice.

## Case presentation

**Figure 1:**
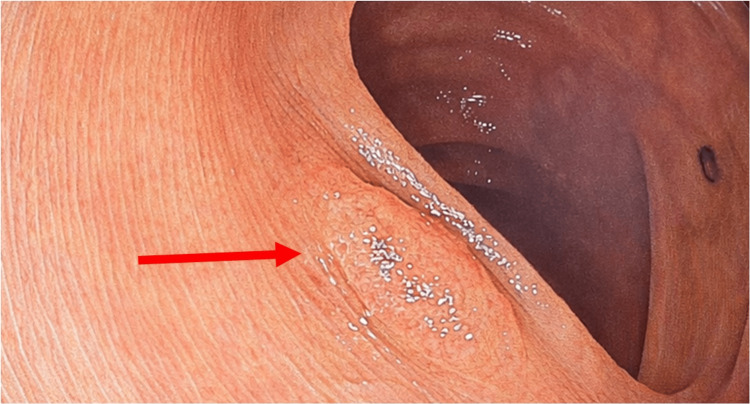
Conventional white-light colonoscopy during routine withdrawal. Representative view of colonic mucosa and a polyp seen during white light inspection (red arrow).

A 67-year-old male underwent an elective colonoscopy for the investigation of a positive faecal occult blood test using AI-assisted CADe. The procedure was completed to the caecum with intubation of the terminal ileum, which appeared macroscopically normal. Bowel preparation was adequate. No macroscopic colitis or diverticular disease was identified. Small internal haemorrhoids were noted. A representative endoscopic view obtained during routine high-definition white-light inspection prior to CADe activation is shown in Figure 1.

**Figure 2:**
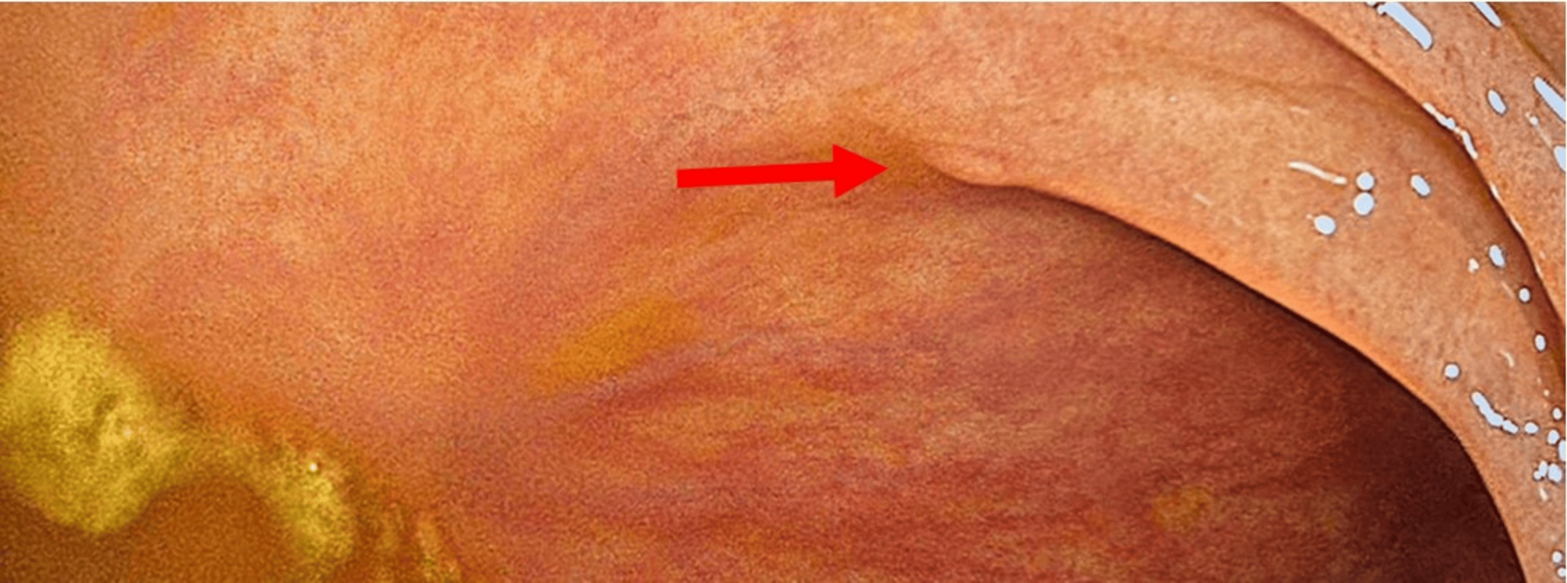
Pre-CADe appearance. Subtle mucosal abnormality under conventional white-light inspection. Retrospective review demonstrates a low flat mucosal irregularity that was not identified during initial white-light inspection (red arrow). CADe, computer-aided detection

**Figure 3:**
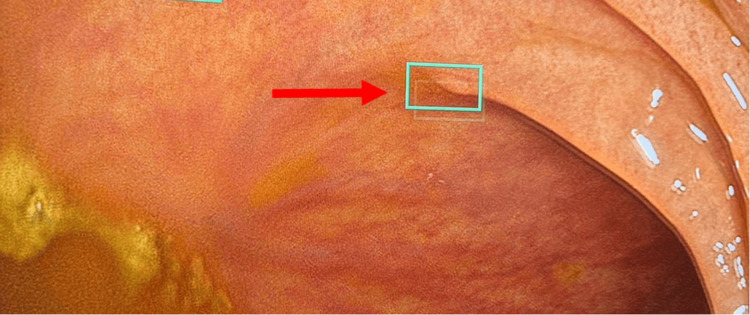
CADe-prompted detection of a subtle lesion with advanced histology. The same mucosal area following real-time CADe prompting, leading to targeted inspection and resection (CADe detection highlighted with a green box, red arrow denotes the subtle lesion). Histopathology demonstrating a tubulovillous adenoma with focal high-grade dysplasia. CADe, computer-aided detection

During a single continuous withdrawal, conventional white-light inspection initially identified six visually apparent polyps. Following activation of the CADe system during the same withdrawal, an additional 30 polyps were highlighted in real time that had not been prospectively recognised by the endoscopist during standard inspection. One such lesion appeared as a subtle flat mucosal abnormality on conventional white-light inspection (Figure 2) and was subsequently highlighted following CADe prompting (Figure 3), leading to targeted resection. Polyps were distributed throughout the colon, including the caecum (n = 4), hepatic flexure (n = 18), transverse colon (n = 5), and sigmoid colon (n = 9).

Polypectomy was performed using a combination of hot and cold snare techniques. There were no immediate procedural complications, including bleeding or perforation. The total withdrawal time was 52 minutes, reflecting extensive lesion detection and resection prompted by AI assistance.

**Figure 4:**
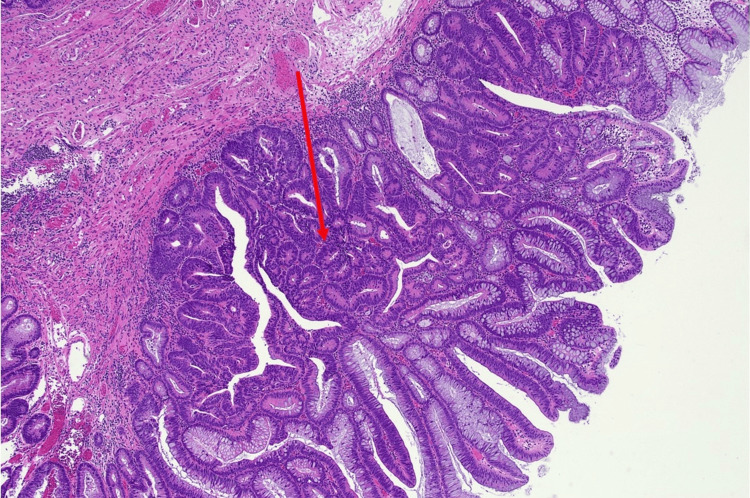
Low-power view (H&E stain, ×40) showing area of high-grade dysplasia (red arrow) with glandular fusion, complexity and hyperchromasia.

**Figure 5:**
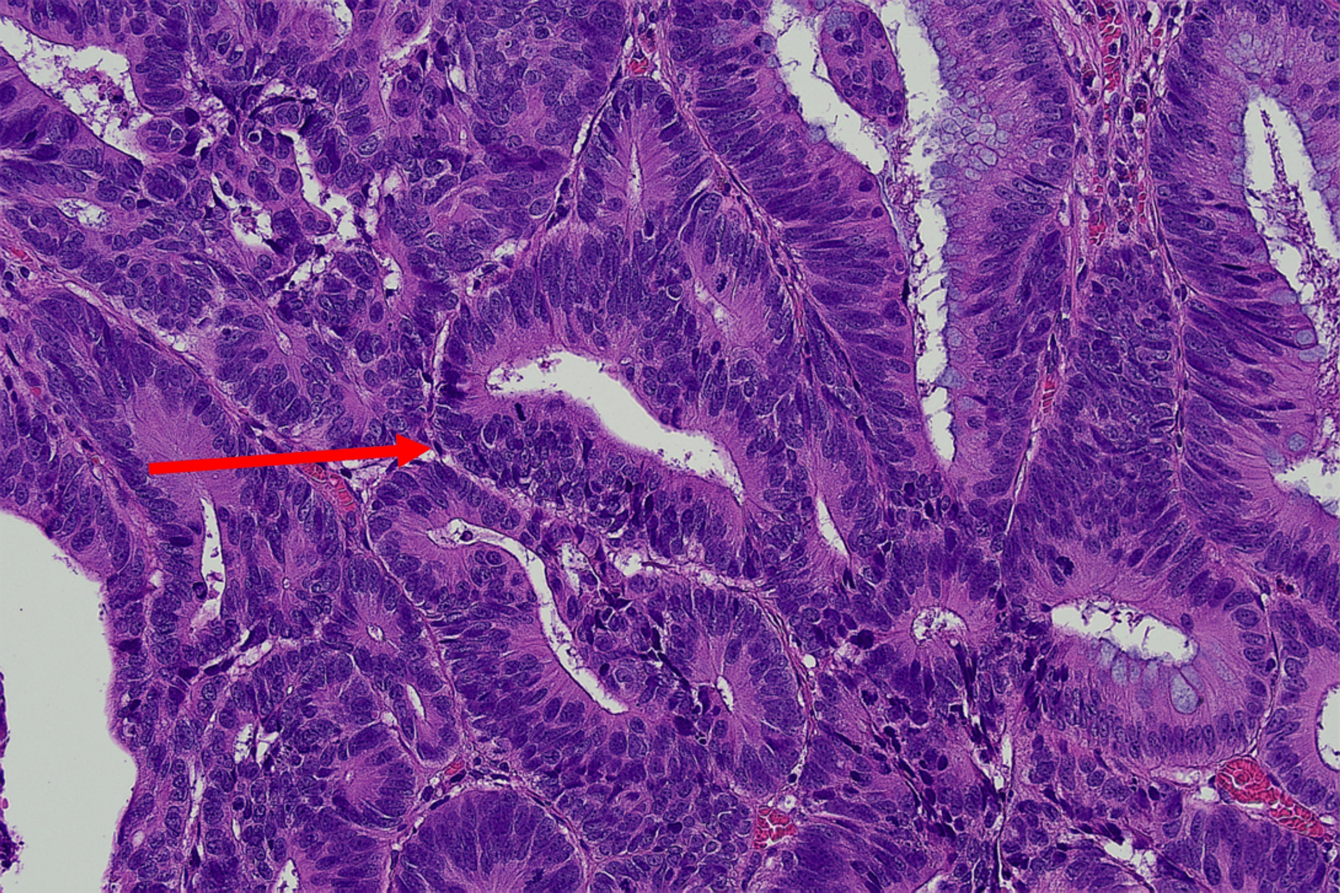
High-power view (H&E stain, ×200) showing markedly atypical architectural and cytological nuclear features with numerous mitoses identified (red arrow).

Histopathological analysis demonstrated predominantly adenomatous pathology. Most lesions were tubular adenomas with low-grade dysplasia. Additional findings included fragments of sessile serrated lesions without dysplasia in the hepatic flexure and a tubulovillous adenoma with focal high-grade dysplasia in the sigmoid colon, corresponding to the CADe-detected lesion shown in Figure 3. No invasive carcinoma was identified. Representative low-power examination demonstrated a region of high-grade dysplasia with glandular fusion and complexity, as well as hyperchromasia (Figure 4). High-power examination revealed markedly atypical architectural and cytological nuclear features with numerous mitoses (Figure 5).

## Discussion

CADe-assisted colonoscopy has been increasingly adopted to improve ADR, a validated quality indicator inversely associated with post-colonoscopy CRC risk [2]. Randomised controlled trials and meta-analyses consistently demonstrate that CADe improves overall ADR and reduces adenoma miss rates, primarily through increased detection of diminutive and subtle adenomas [6-9].

The present case highlights a less frequently examined implication of CADe use: detection and resection of lesions that are not readily appreciable on conventional white-light inspection but are identified following AI prompts. In this patient, several AI-prompted lesions were histologically confirmed as tubular adenomas with low-grade dysplasia, consistent with evidence that most additional CADe-detected lesions are diminutive adenomas rather than advanced neoplasia [7,9,10].

Evidence regarding the impact of CADe on advanced adenomas and sessile serrated lesions remains inconsistent. While some trials report modest increases in advanced adenoma detection, pooled analyses have not consistently demonstrated statistically significant improvements compared with standard colonoscopy [9,10]. In this case, the single tubulovillous adenoma with focal high-grade dysplasia corresponded to a lesion highlighted only after CADe prompting during the same withdrawal (Figure 3). Although it remains uncertain whether this lesion would ultimately have been recognised during continued conventional inspection, its identification was temporally associated with AI prompting, suggesting a potential direct clinical benefit of CADe assistance in this instance.

Of the 30 additional CADe-prompted lesions, 28 were tubular adenomas with low-grade dysplasia, one was a sessile serrated lesion without dysplasia, and one was a tubulovillous adenoma with focal high-grade dysplasia. Importantly, adenomas with high-grade dysplasia are recognised as premalignant lesions, and their removal has been shown to reduce CRC incidence and mortality [3-5]. Although long-term outcome data demonstrating CRC incidence reduction attributable specifically to CADe are currently lacking, detection and resection of adenomas with high-grade dysplasia - established precursors to invasive carcinoma - represent a biologically plausible mechanism by which AI-assisted colonoscopy may contribute to CRC prevention [2,7].

Procedural implications of CADe use are also relevant. While controlled trials report only modest mean increases in withdrawal time, real-world data suggest substantially greater variability [9-11]. In the present case, extensive AI-prompted detection and polypectomy resulted in a withdrawal time of 52 minutes. In practical terms, the detection of one advanced lesion required the resection of 29 additional low-risk lesions, illustrating the trade-off between enhanced detection sensitivity and procedural intensity.

In addition to true-positive detections, CADe systems are known to generate false-positive alerts triggered by non-neoplastic features such as residual faecal material or prominent mucosal folds [9,11,12]. These alerts require active visual confirmation by the endoscopist, potentially interrupting procedural flow, increasing cognitive load, and contributing to prolonged withdrawal time. Beyond procedural efficiency, emerging observational data raise concerns regarding potential behavioural effects associated with routine CADe use. One multicentre study reported reduced ADRs during non-AI colonoscopy following prior exposure to CADe systems, suggesting a possible alteration in visual search patterns [13]. Although causality cannot be inferred, these findings underscore the importance of integrating CADe as an adjunct to - rather than a replacement for - endoscopist expertise and vigilance.

Overall, this case illustrates the dual impact of CADe-assisted colonoscopy: enhanced detection of subtle, histologically neoplastic lesions alongside increased procedural burden and a predominantly low-risk pathological yield. Future research should extend beyond detection metrics to include patient-centred outcomes, cost-effectiveness analyses, and clearer guidance on managing AI-detected lesions with low apparent malignant potential.

## Conclusions

AI-assisted colonoscopy can lower the visual threshold for lesion detection and facilitate resection of histologically neoplastic lesions that may not be readily appreciable on conventional white-light inspection. This case demonstrates that increased detection sensitivity may be accompanied by substantial procedural burden and resection of predominantly low-risk pathology, while still identifying lesions with advanced dysplasia. As CADe adoption expands, defining evidence-based thresholds for intervention and surveillance will be critical to ensure that improved detection translates into meaningful patient benefit without unnecessary overintervention.

## References

[1] Rawla P, Sunkara T, Barsouk A (2019). Epidemiology of colorectal cancer: incidence, mortality, survival, and risk factors. Prz Gastroenterol.

[2] Corley DA, Jensen CD, Marks AR (2014). Adenoma detection rate and risk of colorectal cancer and death. N Engl J Med.

[3] Winawer SJ, Zauber AG, Ho MN (1993). Prevention of colorectal cancer by colonoscopic polypectomy. The National Polyp Study Workgroup. N Engl J Med.

[4] Brenner H, Stock C, Hoffmeister M (2014). Effect of screening sigmoidoscopy and screening colonoscopy on colorectal cancer incidence and mortality: systematic review and meta-analysis of randomised controlled trials and observational studies. BMJ.

[5] Lieberman DA, Rex DK, Winawer SJ, Giardiello FM, Johnson DA, Levin TR (2012). Guidelines for colonoscopy surveillance after screening and polypectomy: a consensus update by the US Multi-Society Task Force on Colorectal Cancer. Gastroenterology.

[6] Joseph J, LePage EM, Cheney CP, Pawa R (2021). Artificial intelligence in colonoscopy. World J Gastroenterol.

[7] Spadaccini M, Menini M, Massimi D (2025). AI and polyp detection during colonoscopy. Cancers (Basel).

[8] Spadaccini M, Troya J, Khalaf K, Facciorusso A, Maselli R, Hann A, Repici A (2024). Artificial Intelligence-assisted colonoscopy and colorectal cancer screening: Where are we going?. Dig Liver Dis.

[9] Makar J, Abdelmalak J, Con D, Hafeez B, Garg M (2025). Use of artificial intelligence improves colonoscopy performance in adenoma detection: a systematic review and meta-analysis. Gastrointest Endosc.

[10] Repici A, Badalamenti M, Maselli R (2020). Efficacy of real-time computer-aided detection of colorectal neoplasia in a randomized trial. Gastroenterology.

[11] Schöler J, Alavanja M, de Lange T, Yamamoto S, Hedenström P, Varkey J (2024). Impact of AI-aided colonoscopy in clinical practice: a prospective randomised controlled trial. BMJ Open Gastroenterol.

[12] Ladabaum U, Shepard J, Weng Y, Desai M, Singer SJ, Mannalithara A (2023). Computer-aided detection of polyps does not improve colonoscopist performance in a pragmatic implementation trial. Gastroenterology.

[13] Budzyń K, Romańczyk M, Mori Y (2025). Endoscopist deskilling risk after exposure to artificial intelligence in colonoscopy - authors' reply. Lancet Gastroenterol Hepatol.

